# U.S. infant mortality, by gestational age, 2015–2023

**DOI:** 10.1038/s41372-026-02646-5

**Published:** 2026-03-30

**Authors:** Elizabeth R. Wolf, Miheret Yitayew, Nayef Chahin, Karen D. Hendricks-Muñoz

**Affiliations:** 1 Children’s Hospital of Richmond at VCU, Richmond, VA USA; 2 Virginia Commonwealth University, Department of Pediatrics, Richmond, VA USA

**Keywords:** Paediatrics, Public health

## Introduction

Resuscitation of extremely premature infants has increased since the 2015 Neonatal Resuscitation Program lowered the viability threshold from 23 to 22 weeks [1]. Care of the extremely preterm infant has improved with standardized medical protocols, multidisciplinary care teams, and greater attention to organ vulnerability. Despite these important advances, little is known about how infant mortality by gestational age has changed in recent years.

## Materials/subjects and methods

We used all-cause infant mortality estimates from the U.S. Centers for Disease Control (CDC) and Prevention–Wide-Ranging Online Data for Epidemiologic Research (WONDER) linked birth-death database [2], which contains birth and death certificate data from all 50 states, Puerto Rico, and the District of Columbia. We used the most recent years (2015–2023) available for all-cause mortality estimates. For specific maternal and infant medical interventions, only 2017–2023 data were available. We used obstetrical estimates of gestational age 22–42 weeks. Confidence intervals were calculated with CDC specifications using relative standard errors for 100 or more deaths and Poisson distributions for 99 or fewer deaths. The project did not meet the Office of Extramural Research definition of human subjects’ research because personally identifiable data were not used. This study followed STROBE reporting guidelines.

## Results

There were 33,795,010 births that met the inclusion criteria, of whom 15,807 (0.05%) were 22 weeks’ gestation and 24,067 (0.07%) were 23 weeks’ gestation (Table 1). Aggregate all-cause infant mortality was 822.9 (95% CI: 803.8–842.0) per 1000 live births for infants of 22 weeks’ gestation and 1.4 (95% CI: 1.4–1.5) per 1000 live births for infants of 40 weeks’ gestation.

**Table 1:** Aggregate all-cause infant mortality by gestational age in weeks, 2015–2023.

Gestational age (weeks)	Deaths	Births	Mortality per 1000 live births (95% CI)
22	13,008	15,807	822.9 (803.8, 842.0)
23	12,830	24,067	533.1 (521.7, 544.5)
24	10,167	31,074	327.2 (319.9, 334.5)
25	7100	34,256	207.3 (202.0, 212.6)
26	5339	38,867	137.4 (133.4, 141.3)
27	4220	45,059	93.7 (90.7, 96.6)
28	3593	55,273	65.0 (62.8, 67.2)
29	3121	62,394	50.0 (48.2, 51.8)
30	3265	86,441	37.8 (36.5, 39.1)
31	3444	106,900	32.2 (31.1, 33.3)
32	3993	166,321	24.0 (23.3 24.8)
33	4190	233,712	17.9 (17.4, 18.5)
34	5884	473,353	12.4 (12.1, 12.7)
35	6038	654,881	9.2 (9.0, 9.5)
36	8780	1,357,701	6.5 (6.3, 6.6)
37	14,593	3,563,819	4.1 (4.0, 4.2)
38	14,728	5,666,253	2.6 (2.6, 2.6)
39	22,284	12,774,949	1.7 (1.7, 1.8)
40	9302	6,460,593	1.4 (1.4, 1.5)
41	2793	1,851,364	1.5 (1.5, 1.6)
42	363	91,926	3.9 (3.9, 3.9)

**Fig. 1:**
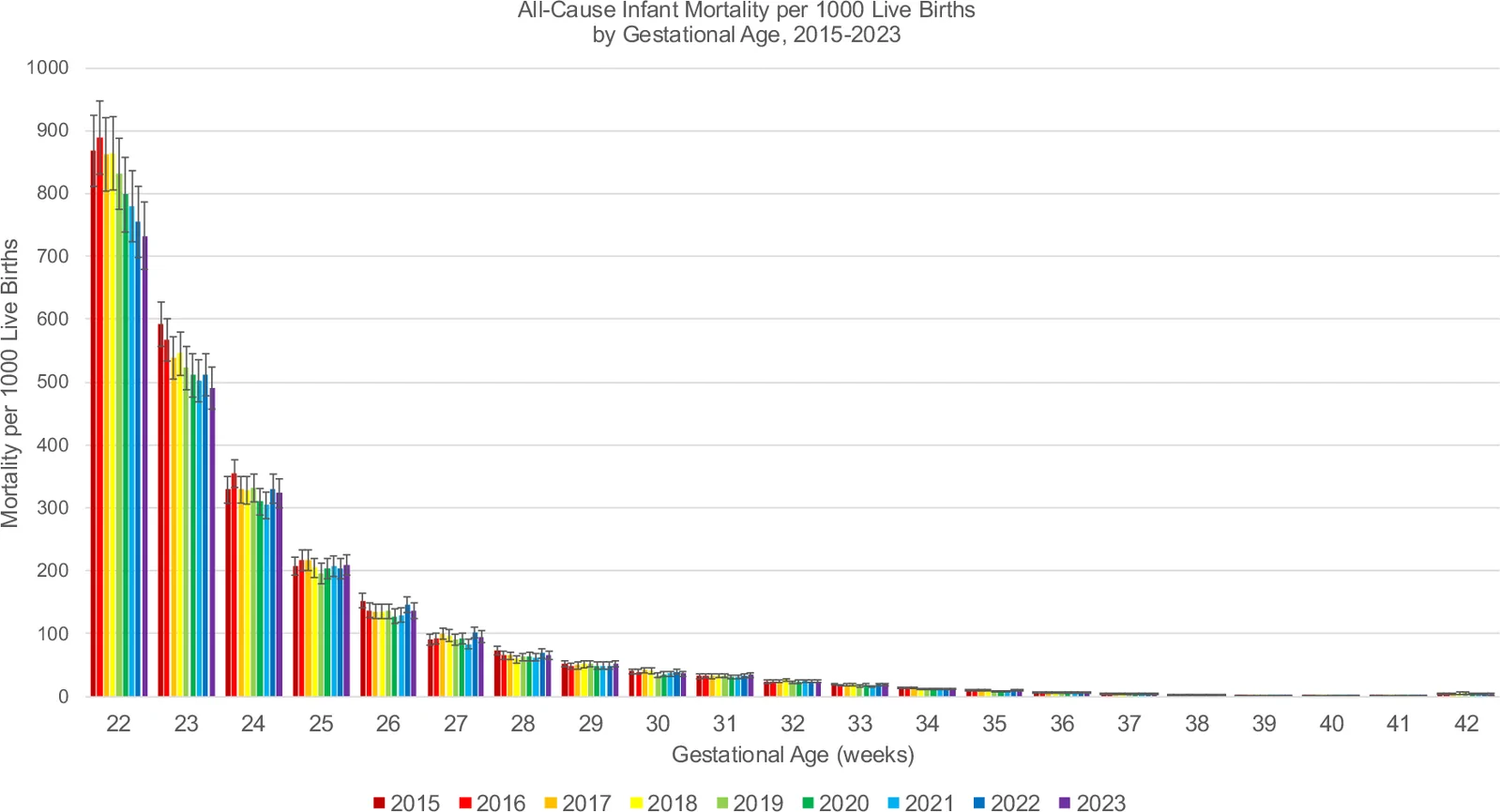
All cause infant mortality per 1000 live births by gestational age, 2015–2023.

Collectively, mortality of infants 22–42-weeks’ gestation declined over the study period from 4.9 (95% CI: 4.8–5.0) per 1000 live births in 2015 to 4.7 (95% CI: 4.6–4.8) per 1000 live births. Mortality of 22-week and 23-week gestation infants declined significantly during the study period (Fig. 1). Mortality for infants 22 weeks’ gestation declined from 867.9 per 1000 live births (95% CI: 811.9–924.0) in 2015 to 732.4 per 1000 live births (95% CI: 678.8–786.1) in 2023. Mortality for infants 23 weeks’ gestation declined from 592.7 per 1000 live births (95% CI: 557.3–628.1) in 2015 to 490.5 per 1000 live births (95% CI: 457.6–523.5) in 2023. Infant mortality at other weeks of gestational age did not change significantly.

For infants of 22- and 23-week gestation, maternal transfer was significantly associated with survival (mortality 563.7 [95% CI: 529.6–597.8] vs. 639.0 per [95% CI: 626.9–651.2] per 1000 live births). Mortality was also lower for infants transferred to another hospital within 24 h compared to non-transferred infants (mortality 518.6 [95% CI: 485.1–552.2] vs. 642.8 per [95% CI: 630.6–654.9] per 1000 live births). Administration of maternal steroids was associated with lower mortality (512.6 [95% CI: 495.9–529.2] vs. 697.5 [95% CI: 682.2–712.8] per 1000 live births). Receipt of surfactants was also associated with lower mortality (476.8 [95% CI: 456.5–497.1] vs. 673.0 [95% CI: 659.4–686.6] per 1000 live births).

## Discussion

We found that mortality for infants of 22–23 weeks’ gestation in the US declined significantly in recent years but remained stable for other gestational ages. Increased survival of extremely premature infants is, in part, driven by the intention to treat. The viability threshold in the US has decreased from 28 weeks in the 1970s to 22 weeks in 2015. The pattern we observed mirrors the improved survival of 24-week gestation infants in the late aughts when resuscitation of these infants became more common. Although the resuscitation of 22-week gestation infants has increased in the US, the practice remains controversial in medical and bioethics forums [3]. The limit of viability in other developed countries varies between 22 and 24 weeks.

Most extreme preterm survivors have major morbidities, including dependence on medical technology. A meta-analysis using studies published between 2000 and 2017 found that of survivors, only 23% of 22-week and 35% of 23-week gestation infants had no neurocognitive impairment [4]. But a recent single-site study suggests that these outcomes may be improving [5]. Nevertheless, growing survivorship of the extremely premature population will likely present challenges to families and healthcare systems in the future.

We found that maternal and infant transfer of 22- and 23- week gestation infants was associated with increased apparent survival. Survival associated with infant transfer is subject to a type of selection bias in which only those who do not die immediately after birth live long enough to be transferred to another facility. The improved survival we observed with surfactant may represent attempted resuscitation rather than medical benefit. More research is needed to understand how to minimize the morbidity of extremely premature infants in the setting of increased survival.

## Data Availability

Data are publicly available from CDC WONDER.
